# Study design and methods for the Breast Cancer and Exercise Trial in Alberta (BETA)

**DOI:** 10.1186/1471-2407-14-919

**Published:** 2014-12-06

**Authors:** Christine M Friedenreich, Sarah MacLaughlin, Heather K Neilson, Frank Z Stanczyk, Yutaka Yasui, Aalo Duha, Brigid M Lynch, Ciara Kallal, Kerry S Courneya

**Affiliations:** Department of Cancer Epidemiology and Prevention Research, Cancer Control Alberta, Alberta Health Services, 1820 Richmond Road SW, Calgary, T2T 5C7 AB Canada; Departments of Oncology and Community Health Sciences, Faculty of Medicine, University of Calgary, Calgary, AB Canada; University of Southern California Keck School of Medicine, Los Angeles, California USA; School of Public Health, University of Alberta, Edmonton, AB Canada; Cross Cancer Institute, CancerControl Alberta, Alberta Health Services, Edmonton, AB Canada; Cancer Epidemiology Centre, Cancer Council Victoria, Melbourne, VIC Australia; Melbourne School of Population and Global Health, Faculty of Medicine, Dentistry and Health Sciences, The University of Melbourne, Melbourne, VIC Australia; Faculty of Physical Education and Recreation, University of Alberta, Edmonton, AB Canada

**Keywords:** Breast cancer, Physical activity, Adiposity, Estrogens, Insulin resistance, Inflammation, Quality of life

## Abstract

**Background:**

Exercise has favorable effects on biomarkers associated with a lower risk of breast cancer, however it is unclear if higher doses of exercise provide additional effects. No clinical trial has systematically examined how different exercise volumes influence the mechanisms underlying breast cancer etiology. The Breast Cancer and Exercise Trial in Alberta (BETA) - a follow-up study to the Alberta Physical Activity and Breast Cancer Prevention (ALPHA) Trial - is examining how a one-year, high versus moderate volume aerobic exercise intervention influences several biomechanisms hypothesized to influence breast cancer risk in a group of postmenopausal women. Secondary aims are to compare intervention effects on psychosocial and quality of life outcomes as well as understand exercise adherence at 12 and 24 months, and maintenance of all study outcomes at 24 months.

**Methods/Design:**

The BETA Trial is a two-center, two-armed randomized controlled exercise intervention trial conducted in 400 previously inactive, postmenopausal women aged 50–74 years, in Alberta, Canada. Participants were randomly assigned to a one-year aerobic exercise intervention of either high volume (300 minutes/week) or moderate volume (150 minutes/week). Blood draws and accelerometry were performed at baseline, six and 12 months. Baseline and 12-month measurements were taken of adiposity (including dual energy X-ray absorptiometry and computed tomography scans), physical fitness, dietary intake, self-reported physical activity and sedentary behavior, quality of life, perceived stress, happiness, sleep, and determinants of exercise adherence. Exercise maintenance was assessed and all study measurements were repeated at 24 months. Blood will be analyzed for endogenous estrogens, insulin resistance indicators, and inflammatory markers.

**Discussion:**

The BETA Trial will compare the impact of a high versus moderate volume of aerobic exercise on a variety of biological, physiological, and psychological outcomes of relevance to postmenopausal women. A tightly controlled exercise intervention and objective outcome measurements are methodological strengths. The BETA Trial will inform future prevention initiatives by assessing adherence to a high volume of exercise over 12 months by postmenopausal women, and the ability of these women to maintain activity over the longer-term. The ultimate objective is to inform public health guidelines for reducing breast cancer risk through physical activity.

**Trial registration:**

Clinical Trials Registration Number: NCT01435005

## Background

Breast cancer is a significant health risk for women in North America, particularly after age 50. Canadian women have a 1-in-9 lifetime probability of developing breast cancer [1] and in the U.S. this estimate is 1-in-8 [2]. Inadequate physical activity is a probable risk factor for postmenopausal breast cancer [3] and is one of the few known risk factors that is modifiable. Recent estimates suggest that physical inactivity accounts for nearly 20% of all postmenopausal breast cancer cases in Canada [4] and about 10% of all breast cancer cases internationally [5]. On average women with the highest versus the lowest activity levels experience a 10-25% lower risk of breast cancer [6, 7]. In postmenopausal women the hypothesized biologic mechanisms underlying the association are not well understood but might involve multiple, interrelated pathways relating to sex hormones, insulin resistance, low-grade chronic inflammation, adipokines and other factors [8] with body fat partially mediating some of these effects [9–12]. As the preventive effects of physical activity become increasingly clear, questions remain surrounding the optimal *dose* of activity that is required to reduce postmenopausal breast cancer risk.

In North America and worldwide, guidelines on physical activity and cancer prevention vary between public health agencies. With respect to aerobic activity, recommendations for adults include:

- ● a minimum of 150 minutes/week of moderate- or 75 minutes of vigorous-intensity activity [13, 14];
- ● 150 minutes/week of moderate-vigorous activity [15, 16]; and
- ● 210 minutes/week of moderate activity [3].

As fitness levels improve, some agencies further recommend:

- ● 300 [13, 14] or 420 [3] minutes/week of at least moderate activity; or
- ● 150 [13] or 210 [3] minutes/week of vigorous activity.

In addition, muscle-strengthening exercise [13, 15] and limiting sedentary behavior [3, 14] are advised. However it remains unproven - and even doubtful [17] - that all of these current recommendations are sufficient for lowering postmenopausal breast cancer risk. These guidelines were based partly on findings from observational studies of physical activity and overall cancer risk. With respect to breast cancer risk specifically, inverse dose–response relations with minutes/week of physical activity have been shown in pooled [18] and meta-analyses [7] of case–control and/or cohort studies. However, observational studies alone cannot inform public health guidelines due to their inherent limitations (e.g., inconsistent definitions of physical activity, physical activity measurement error, and possible confounding by other factors). A more definitive understanding of the dose–response relation can be gained using a randomized controlled trial (RCT) study design.

In the Alberta Physical Activity and Breast Cancer Prevention (ALPHA) Trial [19], we examined how a 12-month aerobic exercise prescription, compared with a usual inactive lifestyle, influenced hypothesized biologic mechanisms for breast cancer risk in 320 postmenopausal women in Alberta, Canada. The exercise group was prescribed 225 minutes/week of moderate-vigorous physical activity, five days a week, with at least half of each workout reaching 70-80% heart rate reserve. Among women assigned to the exercise arm (n = 160), we observed a strong effect of exercise on the main proposed biomarkers of interest that included an effect on endogenous estrogens, insulin resistance, inflammation and body composition [19–22]. In addition, we observed favorable dose–response relations across subgroups of exercise adherence (minutes/week) with respect to 12-month changes in body weight, total body fat, and intra-abdominal fat area [20], circulating free estradiol and sex hormone-binding globulin (SHBG) levels [19], insulin and the homeostasis model assessment of insulin resistance (HOMA-IR), leptin, adiponectin:leptin [21] and high sensitivity C-reactive protein (CRP), with the strongest changes occurring when exercise exceeded 150 or 225 minutes/week. Dose–response relations were not found for androgens [19], tumor necrosis factor-alpha (TNF-α) or interleukin-6 (IL-6) [22]. Furthermore, greater improvements in quality of life variables were observed with exercise duration over 150 minutes/week (p-trend < 0.05 for seven of eight quality of life variables) [23]. Two comparable trials in postmenopausal women [10, 24] observed similar effects of an exercise intervention on hypothesized breast cancer biomarkers as well as some suggestion of a dose–response effect with stronger improvements in some biomarkers when exercise exceeded 195 minutes/week [24–26] or 130 minutes/week [12, 27] but not with others [10, 11, 28, 29]. Yet all of these findings are limited for informing breast cancer prevention guidelines due to the non-randomized, exploratory nature of the analyses (participants within the exercise arm self-selected to adherence levels) and lower statistical power for detecting significant differences in biomarker changes across strata of exercise adherence.

Other RCTs have been designed and statistically powered to compare changes across increasing durations of exercise [30–38] such as the Dose–Response to Exercise in Women (DREW) trial in over 450 postmenopausal women [33, 39–41]. The DREW trial showed favourable dose–response trends across moderate-intensity (50% VO_2max_) exercise durations of approximately 75, 140, and 190 minutes/week and quality of life variables [41] and also weight loss [40], but not for change in waist circumference [40] or CRP levels [39]. While studies like the DREW trial provide more convincing evidence of dose–response relations, the exercise prescriptions did not target primary cancer prevention, few breast cancer biomarkers were examined, and only some of the trials [30, 31, 33, 34] studied postmenopausal women exclusively.

Evolving from the ALPHA Trial, the **B**reast Cancer and **E**xercise **T**rial in **A**lberta (BETA) Trial was conducted in Alberta, Canada between 2010 and 2014. The primary objective was to compare the effects of a high versus moderate volume of aerobic exercise on proposed biologic intermediate endpoints for breast cancer in previously inactive, postmenopausal women. We hypothesized that 300 minutes/week of moderate to vigorous intensity aerobic exercise would induce stronger changes in proposed breast cancer biomarker levels than would 150 minutes/week, the minimum weekly volume that is currently recommended for cancer prevention. Our specific hypotheses were that the higher volume exercise intervention would decrease adiposity levels (body mass index (BMI) (weight (kg)/height (m^2^)), percent body fat, subcutaneous and intra-abdominal fat) and circulating levels of endogenous estrogens (estradiol, estrone, SHBG), insulin resistance indicators (insulin, glucose, leptin, adiponectin), and inflammatory markers (TNF-α, IL-6, high sensitivity CRP) in a dose–response manner compared to the moderate volume exercise intervention. Secondary aims were to evaluate the impact of the intervention on quality of life, perceived stress, happiness, satisfaction with life, sleep quality, exercise adherence, and exercise maintenance 12 months after study completion, and to identify predictors of exercise maintenance. Although we [23, 42, 43] and others [44–46] have assessed these secondary outcomes in previous exercise trials, very few [41, 47] were designed to identify dose–response effects. In addition, we aimed to evaluate the long-term benefit of a higher volume exercise intervention on proposed biomarkers for breast cancer 12 months after study completion. The primary intent of the BETA Trial is to determine whether a higher volume of aerobic exercise is likely to provide further reductions in breast cancer risk compared to the current moderate volume recommendations. Such information will inform public health guidelines addressing how to lower postmenopausal breast cancer risk through physical activity.

## Methods

### Trial overview

**Figure 1:**
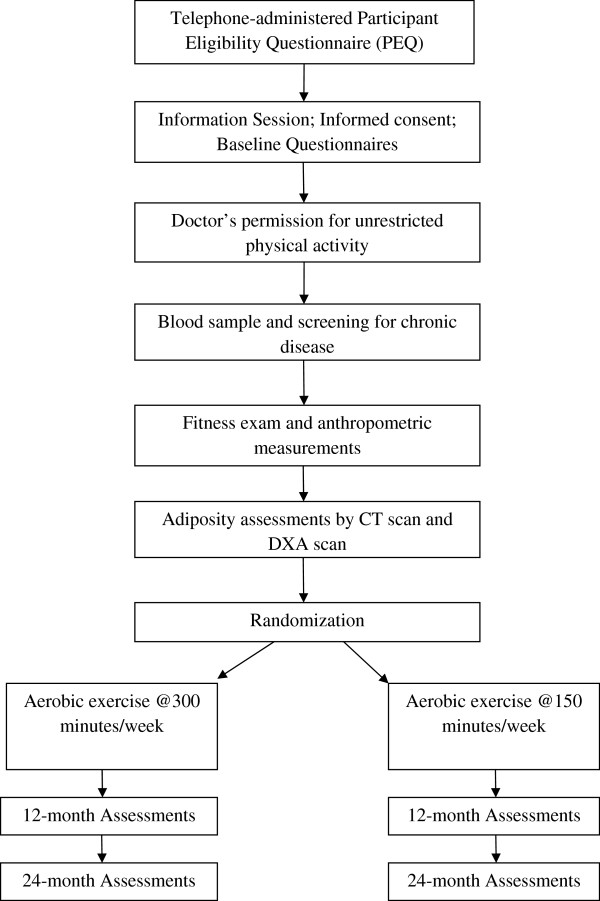
**Participant flow chart for the BETA Trial, Alberta, Canada.**

The BETA Trial is a two-centered, two-armed randomized control exercise intervention trial conducted in Calgary and Edmonton, Alberta, Canada between June, 2010 and June 2014 that included 400 participants. The study protocol was approved by the Alberta Cancer Research Ethics Committee and the Conjoint Health Research Ethics Board of the University of Calgary and the Health Research Ethics Board of the University of Alberta. All participants provided written informed consent. The original study consisted of a year-long intervention with full assessments made at baseline and 12 months. Additional funding was obtained, after the project was initiated, that permitted the expansion of the study to another set of study outcome assessments at 24 months. An overview of participant flow through the study is provided in Figure 1.

### Participants

Several eligibility criteria were used to select the study population, assessed before and after the information session (Table 1). Women were deemed eligible for this trial if they: were 50–74 years of age; postmenopausal; had no previous cancer diagnosis (except for non-melanoma skin cancer) or other major co-morbid condition (e.g. diabetes, cardiovascular disease, arthritis, emphysema) or had recently undergone major reconstructive surgery; were sufficiently physically fit to be able to participate in the exercise program and able to maintain acceptable heart and lung function during a sub-maximal treadmill test; BMI between 22 and 40; moderately inactive as determined by the baseline physical activity and fitness assessments; had no external factors influencing estrogen metabolism, i.e., were non-users of exogenous hormones or drugs related to estrogen metabolism and breast tissue growth; were non-smokers; did not consume more than two drinks of alcohol/day; were English-speaking and able to complete questionnaires and follow instructions in English; were residents of Calgary or Edmonton and able to attend the fitness facility regularly; were not intending to be away for more than four weeks consecutively and eight weeks in total during the year of intervention; and were not currently on a weight loss program or planning to start one.

**Table 1:** **Participant eligibility criteria and assessment methods in the BETA Trial, Alberta, Canada**

Reason for criterion	Assessment methods	Timing of assessment
**Appropriate target group for breast cancer risk reduction**		
Female	*Screen Test* ^*1*^ database	Before contacting participant/immediately upon contact
Age 50–74 years at baseline	*Screen Test* database or PEQ^2^	Before contacting participant
Postmenopausal	PEQ	After participant indicates interest
No previous breast cancer diagnosis	PEQ	After participant indicates interest
**Fit to undertake exercise program**		
No major co-morbidities	Screening questionnaires and blood tests	After participant indicates interest
No previous invasive cancer	PEQ	After participant indicates interest
Passes the self-completed Physical Activity Readiness Questionnaire	PEQ	After participant indicates interest
Obtains physician approval to participate	PARmed-X^3^; approval to do 60 minutes of aerobic exercise (“unrestricted physical activity – start slowly and build up gradually”) five times/week for 12 months	After *Information Session*
Acceptably healthy heart and lung function during fitness test	Sub-maximal treadmill test	After blood sample and obtaining physician approval
**Risk profile amenable to change with exercise intervention**		
Body mass index 22.0-40.0	PEQ (screening) and objective measurement of height and weight	After participant indicates interest (PEQ) and after exercise tester approval (height and weight)
Moderately sedentary lifestyle (no more than 3 days/week of moderate-intensity recreational activity lasting a maximum of <30 minutes/session). Or, no more than 120 minutes of moderate-intensity recreational activity/week.	PEQ (screening) and PYTPAQ^4^ (comprehensive)	After participant indicates interest (PEQ) and at *Information Session* (PYTPAQ)
Estimated VO_2max_ ≤ 34.5 mL/kg/min. If VO_2max_ was between 34.6 and 37.0 mL/kg/min, accelerometer count <10,000 steps/day over seven days.	Sub-maximal treadmill test; seven-day accelerometry (Actigraph GT3Xplus®) if VO_2max_ 34.6-37.0 mL/kg/min	After physician approval
**No other outside factors to influence estrogen metabolism**		
Not currently, previously (<6 months) or planning to take drugs related to estrogen metabolism or breast tissue growth	PEQ	After participant indicates interest
Not a current smoker, or excessive drinker	PEQ	After participant indicates interest
Smoker = needs to have quit for at least 6 months		
Drinking maximum = no more than 14 drinks/week		
Not currently or planning to undertake a weight control/loss program or taking weight loss medications	PEQ	After participant indicates interest
**Logistics**		
Lives in Calgary or Edmonton	*Screen Test* database and PEQ	Before contacting participant and after participant indicates interest
English-speaking	Initial contact with participant and PEQ	After participant indicates interest (telephone recruiters will ascertain)
Not planning to be out of Calgary/Edmonton area more than 4 consecutive weeks during the subsequent 18 months or more than 8 weeks during the entire year.	PEQ	After participant indicates interest

^1^Screen Test: The Alberta Breast Screening Program.

^2^PEQ: Participant Eligibility Questionnaire.

^3^PAR-medX: Physical Activity Readiness Medical Examination.

^4^PYTPAQ: Past Year Total Physical Activity Questionnaire [51].

A number of strategies were used to recruit participants for this study. The primary method of recruitment was through the Calgary and Edmonton centers of *Screen Test*, the Alberta Breast Cancer Screening Program. This method was chosen because it provided access to an appropriate study population that is similar in education and ethnicity to women of the same age in the general Alberta population and had been used successfully previously in the ALPHA Trial [20]. All women in the *Screen Test* database who were between the ages of 50–74, living in Calgary and Edmonton and had attended *Screen Test* for a mammogram within the previous two years, from 2008 to 2010, were sent letters of invitation directly from the *Screen Test* Chief Radiologist. Using *Screen Test* was advantageous because the invitation was endorsed by an authorized custodian of their information and came from an organization in which they already had an established relationship. These letters of invitation were followed up with individual phone calls to determine eligibility and to invite interested individuals to an information session. Up to six phone calls were made to each selected *Screen Test* participant. Additional participant recruitment was done through media campaigns conducted in both cities twice during the trial. These media campaigns were very successful and resulted in a large response. Finally, recruitment was also done in collaboration with physicians who are members of the Alberta Family Physicians Research Practice Network and who agreed to display study materials in their offices and clinics. Brochures and posters were distributed to 90 family physicians in Calgary and Edmonton with information on the study and links to a study website specially designed for this project.

All women who were invited or who contacted our study centers underwent preliminary screening for eligibility (using the Participant Eligibility Questionnaire; see Table 1) by telephone. Women who remained eligible and interested were invited to attend an information session at either the Tom Baker Cancer Center in Calgary or the University of Alberta in Edmonton. At the information session, the study rationale, methods, intervention and follow-up were explained in detail and the roles and responsibilities of the study participants clearly outlined. If these women remained interested in the study, informed consent was obtained and baseline questionnaires were completed at the information session.

The locations of study assessments were as follows. Health-related fitness assessments for Edmonton participants took place at the Cross Cancer Institute and the Behavioral Medicine Fitness Center at the University of Alberta. In Calgary, participants were assessed at the Tom Baker Cancer Center and at the Faculty of Kinesiology, University of Calgary. Full body dual energy X-ray absorptiometry (DXA) scans were taken for Calgary participants at the Human Performance Laboratory, Faculty of Kinesiology, University of Calgary and for Edmonton participants at the Human Nutrition Centre, University of Alberta. Computed tomography (CT) scans were taken at the Foothills Medical Centre in Calgary and at the Cross Cancer Institute in Edmonton. Supervised exercise sessions took place at the Westside Recreation Centre in Calgary and the Behavioural Medicine Fitness Centre at the University of Alberta in Edmonton.

### Randomization

Randomization was stratified by study centre (Edmonton or Calgary) and baseline BMI (< or ≥30) to ensure balance between the two arms with respect to study sites (i.e., potential differences in the participant populations) and the proportion of obese participants. Blocking was used within strata with blocks of four or six to ensure balance between the two arms with respect to the number of participants. The use of different blocks sizes that we randomly permuted made it harder to predict the sequence that was up-coming. The random allocation sequence was generated by user-defined functions in R software (version 2.11). The allocations were concealed in numbered envelopes prepared by a staff member unrelated to the study team. Randomization was performed only for women who were eligible for the study and had completed all baseline measurements (Figure 1). Each participant was randomly assigned to either the HIGH volume (300 minutes/week) aerobic exercise intervention group or to the MODERATE volume (150 minutes/week) aerobic exercise control group. Participants and exercise trainers, by necessity, were unblinded to the intervention assignment.

### Interventions

The two exercise groups were determined by doubling the duration of each exercise session for the HIGH versus MODERATE group. Both groups completed the same frequency (five days/week) and intensity (moderate-to-vigorous) of aerobic exercise. The targets were 60 minutes/session, totaling 300 minutes/week for the HIGH group, and 30 minutes/session, totaling 150 minutes/week for the MODERATE group (Table 2). The volume of 150 minutes/week, the minimum amount recommended for cancer prevention, was based on Health Canada’s Guidelines for aerobic activity [15, 16] and physical activity guidelines from the American Cancer Society [14, 48]. The HIGH group was assigned 300 minutes/week to double the MODERATE prescription. Both groups participated in a structured exercise program consisting of three supervised and two unsupervised sessions/week. Polar® FT4 heart rate monitors (^©^Polar Electro, Canada) were given to each participant and used to ensure a 65-75% maximum heart rate reserve (HRR) was being achieved during each exercise session. The percentage of HRR was chosen because true VO_2_ maximum values were not obtained during the baseline fitness testing, only sub-maximal VO_2_ values. Heart rate reserve, the next most suitable measure, is equivalent to VO_2_ reserve and can be used in exercise prescriptions [49]. The frequency, intensity and duration of exercise were gradually increased during the first three months of the intervention until the target exercise prescriptions were attained (Table 2). This ramp-up period was implemented to reduce the risk of injury and familiarize the previously inactive participants to frequent exercise. Once full prescription was reached, participants were closely monitored through scheduled appointments and direct supervision by a team of exercise trainers to ensure targets were being maintained.

**Table 2:** **Year-long exercise intervention protocol for postmenopausal women in the BETA Trial, Alberta, Canada**

				Duration of exercise in minutes	
Week	Frequency of supervised sessions	Frequency of unsupervised sessions	Intensity (% HRR ^1^ )	High volume group	Moderate volume group
1	3	0	50-60	15 – 20	10 – 15
2	3	0	50-60	15 – 20	10 – 15
3	3	0	55-65	20 – 25	15 – 20
4	3	0	55-65	20 – 25	15 – 20
5	3	1	60-70	25 – 30	15 – 20
6	3	1	60-70	30 – 35	20 – 25
7	3	1	60-70	35 – 40	20 – 25
8	3	1	60-70	40 – 45	20 – 25
9	3	2	60-70	45 – 50	25 – 30
10	3	2	65-75	50 – 55	25 – 30
11	3	2	65-75	55 – 60	25 – 30
12	3	2	65-75	60	30
13-52	3	2	65-75	60	30

^1^% HRR: percentage of calculated heart rate reserve.

Any kind of aerobic activity that raised the participants’ heart rate into their target zone was allowed as part of this exercise intervention. All exercise sessions were recorded by the exercise trainers on weekly exercise logs. For each session, participants reported the date, type of aerobic activities, total exercise time, total amount of time spent in their target heart rate zone, average heart rate as well as their rate of perceived exertion during the session. Beginning at week 5 of the intervention, participants were required to incorporate home-based or unsupervised exercise sessions. For home-based sessions participants recorded their activities in journals that were provided. Unsupervised sessions were also recorded and saved by the participants’ heart rate monitors and documented by an exercise trainer at the next supervised session in weekly exercise logs.

In addition, an educational manual developed by our exercise trainers, who are trained exercise physiologists, was created specifically for this study. The manual included 52 weeks of tips on how to start and maintain an exercise program. Several topics were covered ranging from how to choose appropriate clothing for exercise, how to use the aerobic exercise equipment and avoid injury, and on maintaining interest and enthusiasm in the exercise program. The manual was provided to each study participant during the first exercise session. During that session, the exercise trainer reviewed all of the material in the manual one-on-one with each participant. The exercise program was then individualized to the age, fitness level, interests of each participant. The exercise trainers closely monitored the participants during the first weeks of the exercise intervention to ensure that the participants were able to manage the increased frequency, duration and intensity of the intervention over the three month ramp-up period. They also re-assessed their fitness levels every three months in order to adjust the exercise prescription to ensure that the study participants were continually able to improve their level of energy expenditure at each session.

### Methods to improve study adherence

The exercise intervention was based on the Theory of Planned Behavior [50], which is a motivational theory of human behavior. Various behavior change techniques were incorporated into the intervention to increase intentions to exercise (i.e., motivation to do the exercise program), planning for exercise (i.e., detailed plans for the exercise program), instrumental attitudes (i.e., anticipated benefits of the exercise program), affective attitudes (i.e., anticipated enjoyment of the exercise program), injunctive norm (i.e., perceived approval and social support for the exercise program), descriptive norm (i.e., perceived role modeling of the exercise program), and perceived behavioral control (i.e., perceived controllability and self-efficacy for doing the exercise program).

#### Start Up package, incentives and feedback

Since the participants were inactive when they enrolled in the study, a start up package with various items needed to begin a regular exercise program was provided to each participant. The package included: a voucher for a new pair of running shoes, a water bottle, an athletic t-shirt and a journal to document their weekly activities and progress. Other incentives, such as gift certificates (obtained through donations), were given to participants as they reached various milestones throughout the trial. Participants were provided with continuous feedback on their progress from the exercise trainers and were given a summary and explanation of results from all fitness and anthropometric assessments that were taken at the beginning and end of the study.

#### Educational and group sessions

The exercise trainers facilitated monthly hour-long group exercise sessions for participants. These sessions were an opportunity for participants to learn new aerobic activities and interact with other participants. Educational sessions also instructed participants on various topics related to overall health and wellness, e.g., injury prevention, learn to run, stretching and flexibility. Sessions were not mandatory but participants were encouraged to attend.

#### Adherence challenges

Adherence challenges were used to improve compliance during periods when attendance was expected to be lower than normal, such as the summer months and winter holidays. During these challenges, participants with perfect or near perfect compliance were eligible to win various prizes e.g., gift certificates, donations from local merchants, to acknowledge their commitment to the study.

#### Parking expenses

To ensure participants did not incur additional expenses while participating in the study, any parking costs associated with study-related appointments were reimbursed.

#### Vacation planning

Our exercise trainers met with the participants before planned vacations and completed a *Vacation Planning Form* that included strategies for maintaining exercise whilst away. Since each participant had her own heart rate monitor, they were asked to continue wearing the monitor and maintaining their weekly exercise logs for the duration of their vacation. These materials were returned to study staff at the end of the vacation.

#### Absences and illness

Participants were expected to plan their weekly supervised sessions and to book these with our exercise trainers who followed up by telephone and email with any participants who missed their supervised sessions. Reasons for absences were recorded and a central communication book was maintained so that all exercise trainers knew the status of each participant at all times. If a participant was ill and unable to exercise, the exercise trainers worked with them individually to help them resume their exercise program once they returned to regular sessions.

#### Newsletters and website

To maintain contact with our study participants, we created a quarterly newsletter that was mailed or emailed to all participants directly. It included updates on the study progress, profiles of study participants from each center, study staff profiles, as well as general articles on health topics. We also created and maintained an active study website ( http://www.beta-trial.com) that was used extensively for communication and information dissemination with the study participants. Finally, we produced a cookbook with recipes from study participants and staff that was professionally printed and distributed as a gift and souvenir of the research study to all the participants. Collectively, all of these activities increased the sense of attachment and interest in the study.

#### Social events

To maintain engagement and permit social interaction between the participants, exercise trainers and study staff, several social events were organized that included two formal events held at the universities in each centre, as well as events either at the recreational centre or another location off-site. These events included presentations from the study principal investigators on the study progress and from the exercise trainers on tips for maintaining motivation and interest. In addition, some events included guest speakers, booths by local cancer charities and fitness companies, door prizes, activities to increase awareness and contact between participants as well as fundraisers for local cancer charities. Teams of study participants were also created who trained together and walked in fundraising events for these cancer charities. When all women had completed the 12-month intervention in June 2013, wrap up events were held in each centre to provide an overview of the study for all participants and to encourage them to maintain their current activity levels. Participants who achieved 100% adherence throughout the trial were also recognized at these events.

#### Post-intervention resistance exercise training

The exercise intervention in the BETA Trial was restricted to aerobic exercise only. In response to the expressed interests of our study participants, we developed a short one-day resistance exercise training educational session with accompanying reading materials. Once the participants had completed all of their 12-month tests and assessments, they were free to attend one of these sessions led by one of our exercise trainers in each center. The purpose was to introduce the main principles and components of resistance exercise training. There was no requirement to attend and no additional follow-up provided.

#### Data collection and measurements

A complete data collection schedule is provided in Table 3. All assessments, except the PARmed-X and baseline health questionnaire, were completed in the baseline phase, at the end of the 12-month exercise intervention, and again at 24 months to assess exercise maintenance.

**Table 3:** **Data collection schedule for the BETA Trial, Alberta, Canada**

Assessments	Time (months)			
	−1 to 0 baseline*	6	12 end of study	24 follow-up
**Early screening**				
Participant Eligibility Questionnaire	x			
**Questionnaires**				
Baseline Health	x			
Past Year Total Physical Activity Questionnaire	x		x	x
Canadian Diet History Questionnaire	x		x	x
Quality of Life	x		x	x
Predictors of Exercise Adherence	x		x	x
Psychological Stress	x		x	x
Satisfaction with Life, Happiness	x		x	x
Sedentary Behavior (SIT-Q)	x		x	x
Pittsburgh Sleep Quality Index	x		x	x
Participant Satisfaction with Exercise Intervention			x	
**PAR Med-X** ^**1**^	x			
**Objective measurements**				
Blood Sample	x	x	x	x
Submaximal Fitness Test	x		x	x
Anthropometry (height, weight, waist/hip circumference)	x		x	x
Accelerometry	*x* ^2^	x	x	x
CT Scan^3^	x		x	x
DXA Scan^4^	x		x	x

*All baseline measurements and questionnaires were completed prior to randomization. The exercise intervention began at baseline (time 0).

^1^PAR Med-X: Physical Activity Readiness Medical Examination.

^2^Step count data were retrieved from the accelerometer devices to assess study eligibility for any participant with a VO_2max_ determined to be between 34.6 and 37.0 mL/kg/min.

^3^CT Scan: Computed Tomography Scan.

^4^DXA Scan: Dual Energy X-Ray Absorptiometry Scan.

#### Questionnaires

##### Baseline health

Baseline health characteristics and other demographic information were collected through a self-administered questionnaire that we developed and used previously. Questions were asked regarding marital status, education, employment as well as medical, menstrual and reproductive history. Additional questions addressed history of vitamin, medication and exogenous hormone use.

##### Physical activity and sedentary behavior

Physical activity in the previous 12 months was self-reported at baseline and at 12 and 24 months post-randomization using the *Past Year Total Physical Activity Questionnaire* (*PYTPAQ*) [51]. This questionnaire, developed by our study team, measures all types of occupational, household and recreational physical activity and transportation to perform work as well as the duration, frequency and intensity of these activities. The questionnaire has been shown to have acceptable reliability and validity [51]. All reported activities are coded and converted into MET-hours/week/year using the *Compendium of Physical Activities* developed by Ainsworth and colleagues [52–54]. This measure of total physical activity, which includes activities performed outside the intervention, will be used to characterize our study population.

Similarly, participants were asked at baseline and 12 months to report how much time they spent in sedentary activities over the past year using a sedentary behavior questionnaire called the *SIT-Q*. This questionnaire was developed just prior to the BETA Trial by one of our study investigators (BML) and has since undergone psychometric testing and shown to be a reliable and valid tool for sedentary behaviour assessment [55]. Given that sedentary behavior has been linked to many of the same breast cancer biomarkers as physical activity [56, 57], we will explore in our analyses how sedentary behaviour changes with our exercise intervention.

##### Dietary habits

Dietary intake for the previous 12 months was reported at baseline and at 12 and 24 months using the self-administered Canadian version of the US National Cancer Institute’s *Diet History Questionnaire*[58]. Study participants were asked not to change their dietary intake during the study. Hence, we are interested in verifying if dietary intake was changed since the primary purpose of this study is to examine the independent effect of physical activity on breast cancer biomarkers. In addition, given that energy intake impacts body weight, and because dietary composition might impact proposed biomarkers of risk [59–62], diet will be considered as a potential confounder (although the randomization reduces the chances of this) in our statistical analyses.

#### Blood data

A 60 mL blood sample was taken at baseline after a minimum 10 hour fast and complete abstinence from exercise and alcohol for 24 hours. From the 60 mL, 20 mL were used for eligibility screening to assess for underlying medical conditions that would preclude participation. Screening tests included a complete blood count, lipid panel, fasting glucose, creatinine, alanine aminotransferase and thyroid stimulating hormone. Each was required to be within normal reference ranges for the participant to be eligible for the study. If the woman was under 55 years of age and had not had a bilateral oophorectomy or was uncertain of her menopausal status, a follicle stimulating hormone test was also done. The additional 40 mL of blood from the baseline blood draw was saved for analysis of endogenous sex and metabolic hormones and other biomarkers. At baseline, if the participant did not begin the exercise intervention within eight weeks of the initial blood test, a repeat 40 mL, non-screening blood draw was required to ensure true baseline values of the various biomarkers being examined. Additional 40 mL blood samples were taken at six, 12 and 24 months. A complete blood collection, processing, shipping and storage protocol was developed for this study to ensure standardization of procedures at both collection sites. All blood samples are stored in −86°C freezers in the Alberta Cancer Research Biorepository in Calgary. Freezerworks® Unlimited software (version 6.02) was used to track and manage the location and quantity of all collected samples.

Estradiol and estrone will be measured by radioimmunoassay with preceding organic solvent extraction and Celite column partition chromatography steps. The assay sensitivities are 2 pg/ml and 4 pg/ml, respectively, and the interassay coefficients of variation (CVs) are 9-14%. SHBG, insulin and high sensitivity CRP will be measured by solid-phase, two-site chemiluminescent immunometric assays on the Immulite analyzer (Siemens Healthcare Diagnostics, Deerfield, IL). The assay sensitivities are 1 nmol/L, 2 μIU/ml, and 0.02 mg/dL, respectively, and the interassay CVs are <10%. Glucose will be quantified by a standard analytical procedure using the Vitros Chemistry System. Ten additional biomarkers (from our original study aims and hypothesized post-study funding) will be quantified using three different multiplex assays: the Human Adipokine panel, the Human Circulating Cancer panel and the Human High Sensitivity panel according to the manufacturer’s protocol (Millipore, St. Charles, MO, USA). The assays will be performed at Eve Technologies (Calgary, AB, Canada) using the Bio-Plex™ 200 system (Bio-Rad Laboratories, Inc., Hercules, CA, USA). The Human Adipokine panel assesses adiponectin and resistin with assay sensitivities ranging from 2.2-11 pg/mL, the Human Circulating Cancer panel contains leptin, FGF2, prolactin and VEGF with sensitivities ranging from 3.6-42.8 pg/mL, and the Human High Sensitivity panel contains IL-4, IL-6, IL-10 and TNF-α; with sensitivities ranging from 0.11-1.12 pg/mL. Appropriate quality control samples will be used to monitor the reliability of each assay. Blood samples are batched so that each participant’s baseline, six and 12 month samples are in the same batch and an equal number of MODERATE and HIGH exercise volume bloods are in the same batch. Blind duplicates are included in and between batches to estimate coefficients of variation. All assays will be repeated for the 24-month blood samples. All lab personnel will be blinded to the intervention assignment.

#### Health-related fitness assessments

Only participants who successfully passed the baseline blood screening criteria were invited for health-related fitness assessments (Figure 1) that included resting heart rate and blood pressure assessment, a submaximal fitness test, anthropometric and body composition measurements. All fitness assessments were performed by a Canadian Society for Exercise Physiology Certified Exercise Physiologist (CSEP:CEP®) and one assistant exercise trainer. The same testing protocols and equipment were used at the Edmonton and Calgary study centers and all measurements were taken at baseline and at 12 and 24 months.

##### Resting heart rate and blood pressure

Following standard procedures [63] participants were instructed to sit quietly for five minutes before measurements were taken. Heart rate was monitored using a Polar FT4 heart rate monitor (^©^Polar Electro, Canada). Resting blood pressure was measured using a sphygmomanometer (WelchAllyn®) and stethoscope (3 M™ Littmann®). A blood pressure reading of 144/94 mmHg or lower was required for the participant to undergo the fitness test.

##### Body composition/Anthropometry

Anthropometric data on standing height, weight, waist and hip circumference were measured using standardized methods and equipment at the time of the fitness tests. Waist circumference was measured using the National Institutes of Health protocol and hip circumference was measured using the World Health Organization procedure with an anthropometric measuring tape [64]. Full body dual energy X-ray absorptiometry (DXA) scans were taken using a Hologic DXA system in Calgary and a General Electric Lunar iDXA in Edmonton to assess overall percent body fat, total lean body mass, total fat mass and bone mineral density. Computed tomography (CT) scans were taken at the level of the umbilicus to measure subcutaneous and intra-abdominal adiposity. Three single slices were taken at the L5 vertebrae and these data were then transferred to our study radiologist at the Cross Cancer Institute in Edmonton who reviewed each scan individually using a software program (Aquarius Intuition by Terarecon, Inc.) that was used to quantify amount of subcutaneous and intra-abdominal fat at this level of the abdomen. Because of the high cost associated with both the DXA and CT scans, only participants who had completed all screening stages and considered eligible for the study were given these scans at baseline. These assessments were repeated 12 months and 24 months later (Table 3).

##### Cardiorespiratory fitness

All participants completed a sub-maximal cardiorespiratory testing to assess their aerobic fitness and determine eligibility (Table 1; Figure 1). The multistage, modified Balke treadmill test protocol was used [65] in which a speed of 3.0 mph is maintained throughout the test and the grade is gradually increased every three minutes by 2.5% starting at 0%. The test was completed when the participant finished the stage in which she reached 85% of her age predicted maximum heart rate or reached volitional exhaustion. The participant’s predicted VO_2_ max was estimated using the multistage model and the American College of Sports Medicine metabolic equations for estimating maximum oxygen consumption [66]. To be eligible for the BETA Trial, a minimum of two stages of the test had to be completed and estimated VO_2_ max had to be ≤ 34.5 mL/kg/min (Table 1). If the estimated VO_2_ max was between 34.6 mL/kg/min and 37 mL/kg/min, additional assessments of their activity levels were needed. In these individuals additional step count data were retrieved from the accelerometer devices (Actigraph GT3X + ® and *activPAL™*) that all participants were required to wear for seven days. If it was determined that, on average, these participants walked <10,000 steps/day they were eligible for the study. Any woman with an estimated VO_2_max greater than 37 mL/kg/min was ineligible for the study. A modified version of the fitness test was repeated at 3, 6, and 9 months post-randomization for external verification of reported adherence to the exercise prescription and as a means to adjust the prescription. Randomization into the study occurred after all health related fitness tests were completed including the DXA and CT scans (Figure 1).

#### Objective measurements of physical activity and sedentary behaviour

An accelerometer and inclinometer were worn by participants at four time points during the trial - baseline, six, 12 and 24 months (Table 3) - to complement self-reported physical activity and sedentary behavior in the *PYTPAQ* and *SIT-Q*, respectively. The ActiGraph GT3X + ® accelerometer (Actigraph, LLC, Pensacola, FL) was used to assess moderate-vigorous physical activity since the GT3X+ model is particularly suitable for measuring upright moderate-vigorous intensity movement. The accelerometers were worn on the hip and recorded the duration, frequency and intensity of movement. The *activPAL™* inclinometer (PAL Technologies Ltd, Glasgow, Scotland) was adhered to the upper thigh area. This device measured duration and frequency of time spent sitting, standing and stepping (light ambulation), and number of postural changes. The *activPAL™* inclinometer is considered the most accurate device for assessing sedentary time in adults [67]. All participants were asked to wear both devices simultaneously for seven days immediately after their fitness tests. As mentioned above, the step counts on the Actigraph GT3X + ® were also used to determine eligibility in any woman with an estimated VO_2max_ that was deemed potentially too high. Additionally, they were required to record, in a daily activity log, when the devices were worn each day and which activities they were doing when not wearing the device, e.g., showering or swimming since only non-sleep activity was recorded.

#### Participant-reported outcomes

Participants completed self-administered questionnaires at baseline, 12 months, and 24 months to assess quality of life, sleep quality, perceived stress, satisfaction with life, and happiness; psychosocial factors that have been associated with exercise in some intervention trials [23, 44–46] and observational studies [68, 69] and therefore might improve with increasing exercise dose. Quality of life was measured using the *RAND SF-36*[70] which includes 36 questions and uses eight subscales to assess physical and social functioning, pain, mental health. Questions were also asked about stress using the *Perceived Stress Scale*[71] and sleep using the *Pittsburgh Sleep Quality index*[72]. Happiness and satisfaction with life were assessed using the Happiness Measure [73] and the Satisfaction with Life Scale [74], respectively.

#### Determinants of physical activity

The social cognitive determinants of physical activity were assessed at baseline, 12 months, and 24 months through participant-administered questionnaires that followed the standard questions based on the Theory of Planned Behavior [50]. This theory assesses attitude, subjective norm, perceived behavioral control, intentions and planning. At the end of the study, the participants were also asked to complete a *Participant Satisfaction Questionnaire* to assess their attitudes and barriers towards exercise.

#### 24-month follow-Up

An earlier follow-up of ALPHA Trial participants, on average 12 months post-study completion, demonstrated that a high follow-up response rate was attainable; however, this follow-up was restricted to completion of one self-administered questionnaire pertaining to exercise maintenance. In the BETA Trial, 24-month assessments included all questionnaires, the blood draw, fitness tests, anthropometric and body composition assessments and objective measures of physical activity and sedentary behaviour.

### Sample size

Sample size calculations were based on a standard two-sample mean comparison formula using α = 0.05 (two-sided). Taking a conservative approach, the means of 12-month outcomes (log-transformed, with adjustment for baseline values) were compared. Results from the ALPHA Trial provided estimates of standard deviations and ‘yardstick’ intervention effects that might be expected. A sample size of 150 participants/group was chosen, providing 80% power to detect anticipated changes of 12% in insulin resistance and inflammatory outcomes (leptin, insulin, adiponectin) at 12 months and 99% power to detect changes as low as 2% in all body fat outcomes. While an initial group size of 165 was planned, because of more rapid recruitment than anticipated due to tremendous participant interest in the study, we increased the sample size to 400 per group without incurring extra resource requirements. The increase in sample size created a slight improvement in power, on average, and adequately accounted for an anticipated 10% loss-to-follow up and more modest exercise adherence that we postulated might occur with the higher demands associated with the exercise intervention in the HIGH volume arm of the trial.

### Statistical methods

The trial analysis will follow the intention-to-treat principle, as such all participants who were randomized (n = 400) will be included regardless of their adherence to the study. After initial descriptive and graphical examination of the response measurements (with a consideration of transformation), we will examine correlations among markers within each of the hypothesized pathways that are being examined between physical activity and breast cancer risk. This approach will guide us to a hypothesis-driven pathway-based evaluation of intervention effects. Specifically, the correlation analysis will explore if consistent patterns supportive of a simultaneous evaluation of multiple markers in each pathway is warranted. As a formal evaluation approach, we will apply the linear mixed effects model methodology to compare the changes from the baseline to 12-month follow-up in levels of estrogens, markers of obesity, insulin resistance and inflammation and also in our psychosocial outcomes of interest. The mixed model evaluation will be considered for both individual markers (univariate outcomes) and pathway-based multiple markers (multivariate outcomes), of which we propose the latter to be our primary evaluation scheme.

The analysis will be performed with and without covariate adjustments, initially without any adjustment based on the randomized design, followed by adjustment for covariates in which substantial imbalances between the two groups were observed. Rates of recruitment and adherence to the intervention as well as maintenance of exercise behaviour will be estimated and examined in association with the study participant characteristics.

## Discussion

The primary results from BETA Trial will be central for determining if 300 minutes/week of vigorous intensity aerobic exercise is better than 150 minutes/week for postmenopausal breast cancer risk reduction. Furthermore, any dose–response effects that are found between exercise dose and blood biomarker changes will help to strengthen (or refute) current hypotheses surrounding the biologic mechanisms underlying the association, which remain unclear. Yet the value of the BETA Trial findings depends, first and foremost, on the quality of our study design and data collection methods.

The design of the BETA Trial was informed in large part from our previous experience with the ALPHA Trial in which we found that aerobic exercise influenced several biomarkers associated with breast cancer risk. In addition, we noted a possible dose–response effect between increasing adherence to the exercise intervention and corresponding changes in biomarker levels. Since these comparisons were made within an allocation arm of a trial, rather than from a true randomized comparison, the BETA Trial was necessary to confirm these dose–response effects with prescribed aerobic exercise.

Based on the experience of the ALPHA Trial, we recognized that women willing to participate in an exercise intervention trial strongly preferred being randomized to an exercise group rather than a control group that did no exercise. Furthermore, there was some contamination within the control group of the ALPHA Trial with some study participants starting an exercise and weight loss program on their own. Amongst the control group, 53% started exercising and 63% decreased their caloric intake over the year based on self-report as compared to their baseline assessments of the past 12 months. Given that the relevant research question after the ALPHA Trial was “what is the optimal dose of aerobic activity?” rather than “does exercise influence these biomarkers for breast cancer?”, we opted for a dose–response trial.

Several aspects of the BETA Trial design, including subject recruitment, data collection methods, exercise intervention, sample size and analysis also derived from our experience with the ALPHA Trial. The ALPHA Trial had very high retention and adherence rates and provided meaningful insights into the potential etiology between physical activity and breast cancer risk. Hence, for the BETA Trial, we used the same study methods with some refinements. For example, we recognized the advantage of media campaigns for recruitment and so used these effectively once we had all protocols in place and had begun recruitment through letters of invitation with the breast screening program. The media campaigns provided a large influx of possible participants all at once which demanded an increase in study personnel to meet this recruitment drive. In addition, we created and used a study website more routinely to communicate with our study participants. We also enhanced our newsletters by making them longer, with more information and articles to interest the participants. In the ALPHA Trial, we used group sessions offered every three to four months in addition to individual exercise sessions whereas in the BETA Trial, group exercise sessions were offered every month. We also increased the number of exercise trainers and the facilities were open six days/week with availability 12 hours daily for participants to drop in. In the BETA Trial, we continued the use of several incentives plus additional incentives to enhance adherence, e.g., adherence challenges.

A key component to the success of a multi-centered trial, such as this one, is standardization of all study methods across study sites. Hence, we developed standardized protocols for all of the study methods and a lengthy manual of operations. We held weekly meetings of all team members at both sites that included the exercise testers, study staff and the two principal investigators. In addition, our study staff and exercise testers met throughout the study to ensure that the methods for exercise testing, for delivering the exercise intervention, and for collecting and managing all the data were standardized. Any changes to study procedures or protocols that were needed were fully discussed and implemented at both sites. Several additional factors were crucial to the success of our study. The BETA Trial methods had been largely developed and tested in the ALPHA Trial and therefore we were able to learn from our successes and build upon them to deliver an even better intervention to a larger study population. Recruitment to the study was greater than expected partly because of the heightened awareness of our research activities due to previous publicity from the ALPHA Trial. Also retention to the study was very high in (96%) since we implemented protocols to follow-up our non-adherent participants and provided participants with a number of incentives and feedback on their progress - e.g., receiving results from their health-related fitness and body composition measurements at 12 and 24 months - to help maintain involvement in the trial. We had preliminary evidence from the ALPHA Trial that previously inactive, postmenopausal women could be motivated to achieve and sustain high levels of activity and hence used all of our previously developed approaches, and several new incentives that were outlined above, to achieve exercise adherence even with the HIGH volume group. Another strength of our study was the use of rigorous measurement methods for all of the assessments made, including objective assessments of physical fitness, physical activity, sedentary behaviour and body composition. Our laboratory assays are being conducted in highly reputable laboratories (of co-author FZS) employing methods that provide accurate and reliable measurements.

The implications of the BETA Trial findings are diverse. By prescribing a higher dose of exercise than in previous, comparable biomarker trials [10, 24, 33, 75, 76], the BETA Trial will reveal whether or not greater reductions in proposed biomarkers for breast cancer are possible with additional exercise. Furthermore, further exploration of the mechanisms underlying biomarker changes - e.g., the potential benefit from exercise in the absence of significant weight loss - will enable clearer public health messaging for primary cancer prevention. Our secondary outcomes from the trial, including quality of life, perceived stress, sleep, and happiness, could help motivate inactive women to become physically active after menopause. Furthermore, the BETA Trial will inform future disease prevention initiatives by demonstrating the ability of these women to *achieve* a high volume of regular exercise over the long-term and to *maintain* this level of activity independently, 12 months beyond the intervention period. Ultimately our findings will help to refine and strengthen the current public health guidelines for primary breast cancer prevention, to decrease the future incidence of breast cancer.
